# Identification, Molecular Cloning of IL-1β and Its Expression Profile during *Nocardia seriolae* Infection in Largemouth Bass, *Micropterus salmoides*

**DOI:** 10.3390/ijms17101670

**Published:** 2016-10-01

**Authors:** Ping-Yueh Ho, Omkar Byadgi, Pei-Chyi Wang, Ming-An Tsai, Li-Ling Liaw, Shih-Chu Chen

**Affiliations:** 1 Department of Veterinary Medicine, College of Veterinary Medicine, National Pingtung University of Science and Technology, Pingtung 912, Taiwan; pyho@mdu.edu.tw (P.-Y.H.); omkarcof1@gmail.com (O.B.); william878588@gmail.com (M.-A.T.); 2 Bioresource Collection and Research Center, Food Industry Research and Development Institute, Hsinchu 300, Taiwan; lll@firdi.org.tw

**Keywords:** IL-1β, largemouth bass (*Micropterus salmoides*), *Nocardia seriolae*, immunoadjuvant

## Abstract

In the present study, IL-1β cDNA was identified and analyzed from largemouth bass (*Micropterus salmoides*). Full length IL-1β mRNA was obtained using Rapid Amplification of cDNA Ends (RACE), which contains 78 bp 3′-UTR, a 455 bp 5′-UTR, and an open reading frame (ORF) of 702 bp coding for 233 amino acid residues. The molecular weight and theoretical isoelectric point of largemouth bass IL-1β protein was predicted to be 26.7 kDa and 6.08 respectively. A largemouth bass IL-1β phylogenetic analysis showed a close relation to the IL-1βs of striped trumpeter (*Latris lineata*), Chinese perch (*Siniperca chuatsi*), and Japanese sea bass (*Lateolabrax japonicus*). Peptidoglycan upregulated IL-1β in the spleen and head kidney, while lipopolysaccharide upregulated detectable levels of IL-1β in the spleen only. Largemouth bass, challenged with *Nocardia seriolae* (1.0 × 10^6^ cfu/mL), showed a significant increase in IL-1β at 3 and 5 days post infection (dpi) in the spleen, while in the head kidney significant expression was found at 2 and 3 dpi, peaking at 3 dpi. Furthermore, tumor necrosis factor α (TNF-α) showed significantly higher expression in the spleen at 3 and 5 dpi, and in the head kidney at 1 and 3 dpi, with expression decreasing at 5 dpi in both tissues.

## 1. Introduction

Interleukin-1β is a member of the interleukin-1 family of cytokines that mediates the response of the host to microbial invasion, inflammation and immune reactions. IL-1 has a broad spectrum of physiological and immunological functions, which reflect some of its earlier names, such as endogenous pyrogen, leukocytic endogenous mediator, lymphocyte activating factor, mononuclear cell factor, catabolin, osteoclast-activating factor, and hemopoietin 1 [1]. Eleven members of the IL-1 family (IL-1α, IL-1β, IL-1 receptor antagonist, IL-18, IL-33 and IL-1F5–10) have been identified in mammals [2]. Of these, IL-1β is a proinflammatory cytokine which has a crucial role in regulating the defenses of the host during infection [3].

In mammals, IL-1β is produced as an inactive precursor (proIL-1β) and proteolytically cleaved by the specific IL-1 converting enzyme (ICE or caspase-1) at Asp-X (where X is a small hydrophobic residue) to form a biologically active mature peptide [4,5]. Even though several hypotheses have been proposed on the mechanism by which IL-1β is released into the extracellular space, the secretion through the conventional ER-Golgi route remains elusive [6]. The active IL-1β binds to the type I IL-1 receptor (IL-1RI), which is then followed by the recruitment of receptor-associated protein (IL-1RAcP), and then initiates the signaling pathway. Subsequent combinatorial phosphorylation and ubiquitination activates the nuclear factor κB signaling (NF-κB), and the c-Jun N-terminal kinases (JNK) and p38 mitogen-activated protein kinase (MAPK) pathways, inducing the expression of genes that include IL-6, IL-8, MCP-1, COX-2, IκBα, IL-1α, IL-1β and MKP-1 [7].

IL-1, like bioactivity in teleost fish, was first identified in supernatants from lipopolysaccharide LPS-stimulated monocytes of channel catfish (*Ictalurus punctatus*). Substances within the supernatants caused the mitogen-induced proliferation of, and antigen-induced antibody production by, lymphocytes [8]. Common carp (*Cyprinus carpio*) macrophage, neutrophils, epithelial and macrophage cell lines have also been found to secrete IL-1 like factors [9,10,11]. Fish IL-1 exhibits similarities with its mammalian counterparts, IL-1α and IL-1β, suggesting that it is highly conserved [12]. Despite the importance of IL-1 in the teleost immune system, the fish IL-1β coding gene was not resolved until 1999 [13]. Fish IL-1β transcripts are typically discovered by suppression subtractive hybridization (SSH) or homology cloning [12]. IL-1β has been discovered in a variety of fish with representatives from Salmoniformes, Cypriniformes, Perciformes and Pleuronectiformes. Several studies have shown that fish IL-1β proteins are involved in regulating the inflammatory response to bacterial or parasitic infection [14,15,16,17,18]. However, the largemouth bass IL-1β (LMBIL-1β) has not been isolated, nor has its expression in response to pathogens infecting the largemouth bass been characterized.

*Nocardia seriolae* is the etiological agent of fish nocardiosis [19], which is characterized by the formation of abscesses on the epidermis and granulomas in the gills, kidney, and spleen, and is a serious disease for cultured yellowtail *Seriola quinqueradiata* and amberjack *S. aureovittata* [20]. It had been diagnosed in disease outbreaks in a range of fish species, including freshwater [21,22,23,24] and seawater fish [25,26,27]. The pathological process of these diseases is characterized by the chronic and slow exhaustion of the infected fish. Also, the evidence of the intracellular *N. seriolae* infection in eliciting inflammation of the fish immune system has seldom been presented. Therefore, in this study, the expression profiles of IL-1β in largemouth bass *Micropterus salmoides*, were investigated during an experimental *N. seriolae* infection. Also, we evaluated an important component tumor necrosis factor α (TNF-α), which can be secreted by different cell types through the stimulation with inflammatory mediators, or cytokines such as IL-1β.

## 2. Results

### 2.1. Cloning and Analysis of Full-Length IL-1β mRNA

**Figure 1:**
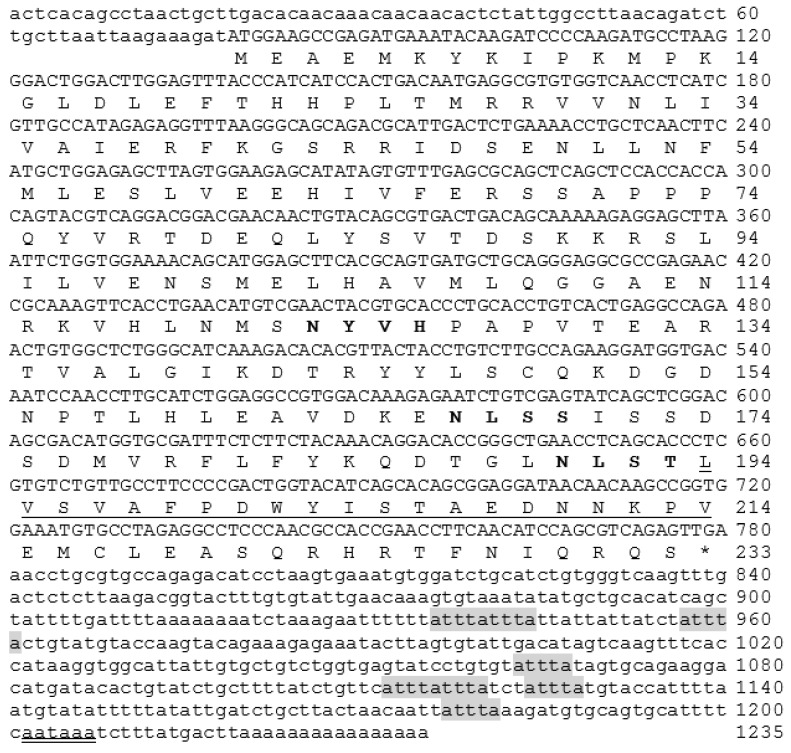
Full-length cDNA of largemouth bass *Micropterus salmoides*, IL-1β. The underlined sequence part is IL-1β signature and grey highlighted sequences are ATTTA motifs, bold letters are *N*-glycosylation sites and double underlined are AATA motif.

A PCR-amplified fragment of largemouth bass IL-1β was produced using degenerated primers, with an amplicon of 515 bp. The 5′ Rapid Amplification of cDNA Ends (RACE) by primer LMBIL-1β3R1 and universal primer mix (UPM) yielded a 680 bp fragment, while the 3′ RACE produced a 745 bp fragment. These fragments were overlapped and subtracted to yield a final sequence of a 1235 bp IL-1β transcript (Figure 1). A full-length mRNA containing a 78 bp 5′-UTR and a 455 bp 3′-UTR, and an ORF of 702 bp coding for 233 amino acid residues, was obtained. An IL-1β signature was found between amino acids 194 and 214 in the deduced peptide. Three possible *N*-glycosylation sites at amino acid positions 123, 167 and 190 were postulated. The 3′ UTR contained eight unstable motifs (ATTTA) and a polyadenylation signal of 18 bp upstream to the poly A tail (Figure 1).

**Figure 2:**
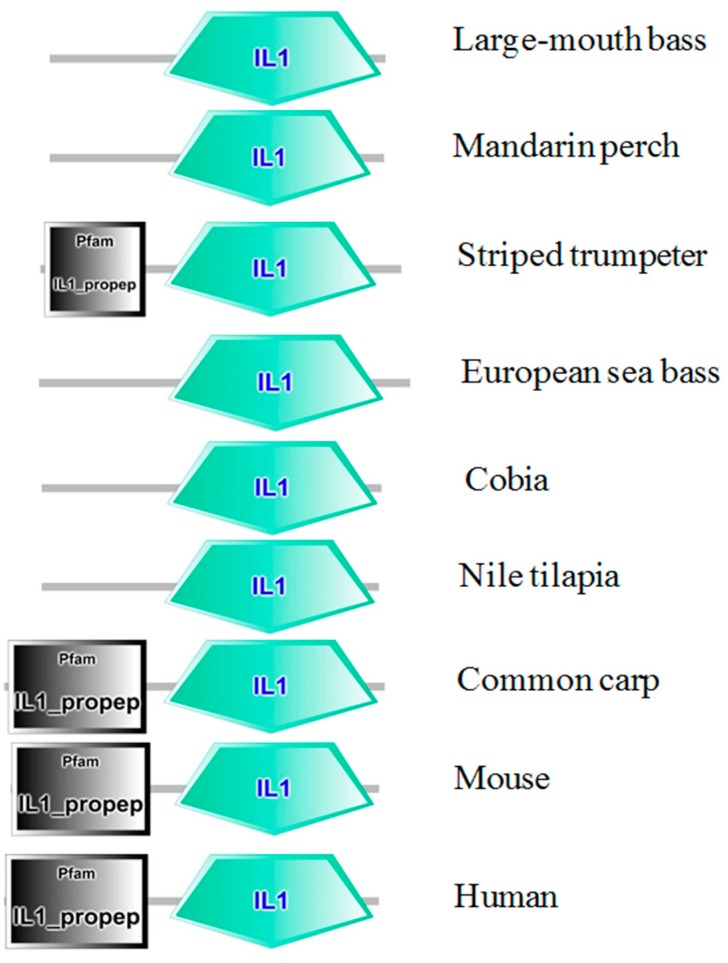
IL-1β domains in various fish species, mouse and human. The IL-1β comparison of structure were predicted by SMART analysis of the amino acid sequence from Mandarin perch (AAV6501.1), Striped trumpeter (ACQ99510.1), European seabass (CAC41006.1), Nile Tilapia (XP_003460673.3), Common carp (BAA24538.1), mouse (AAA39276.1) and human (AAA59135.1).

**Figure 3:**
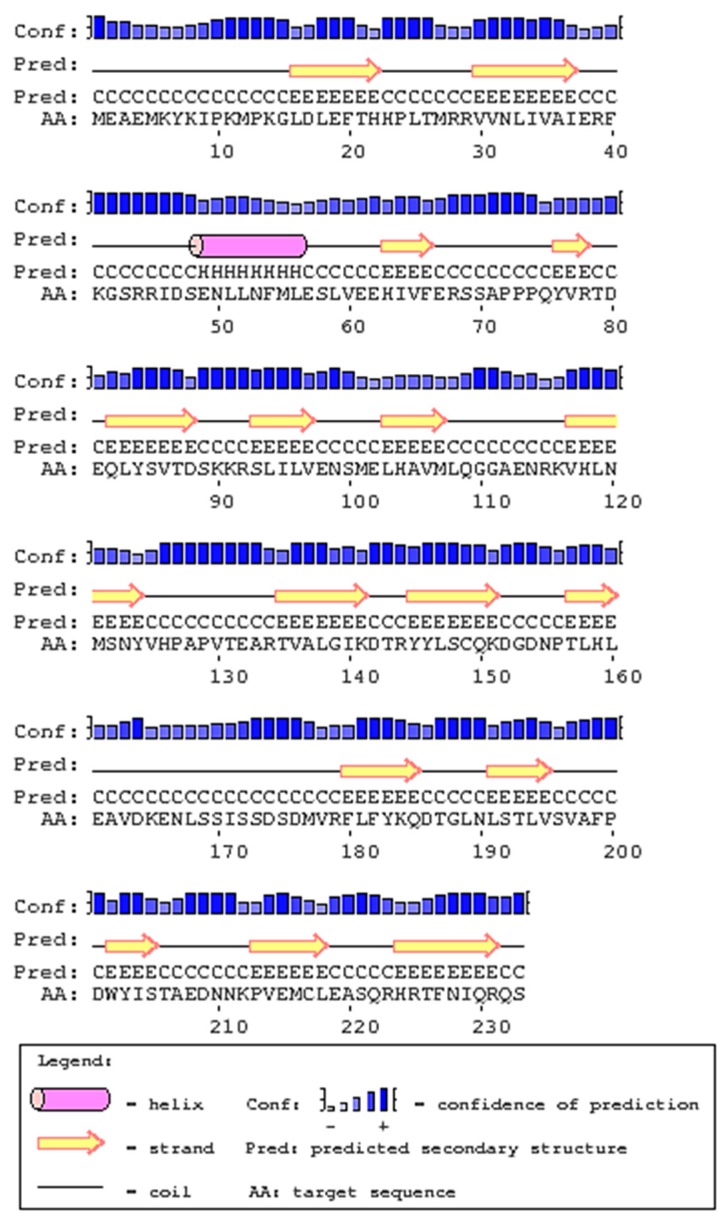
Predicted secondary structure of IL-1β protein containing one helix shown as a cylindrical structure.

**Figure 4:**
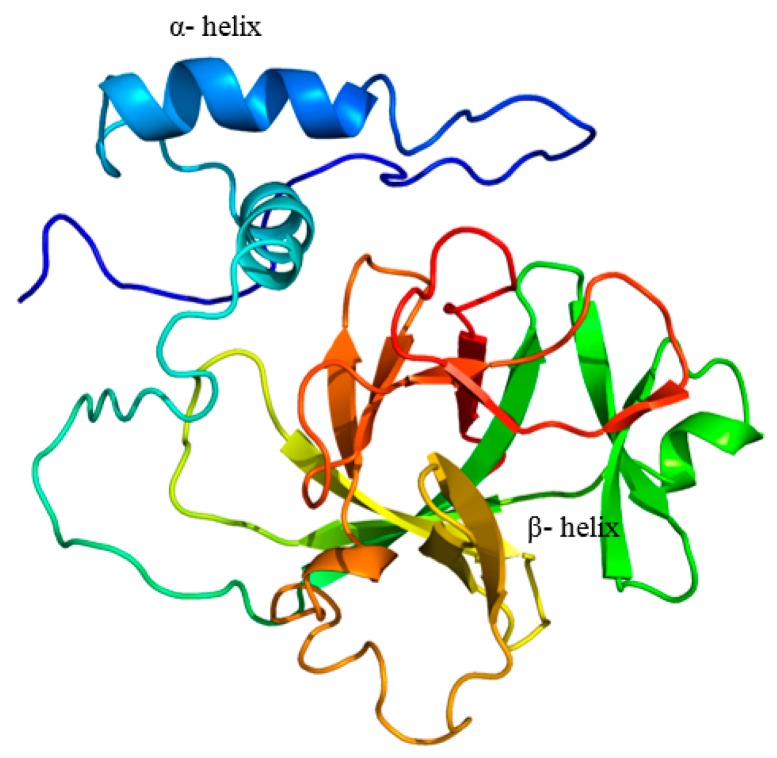
Predicted 3D structure of largemouth bass IL-1β protein.

**Figure 5:**
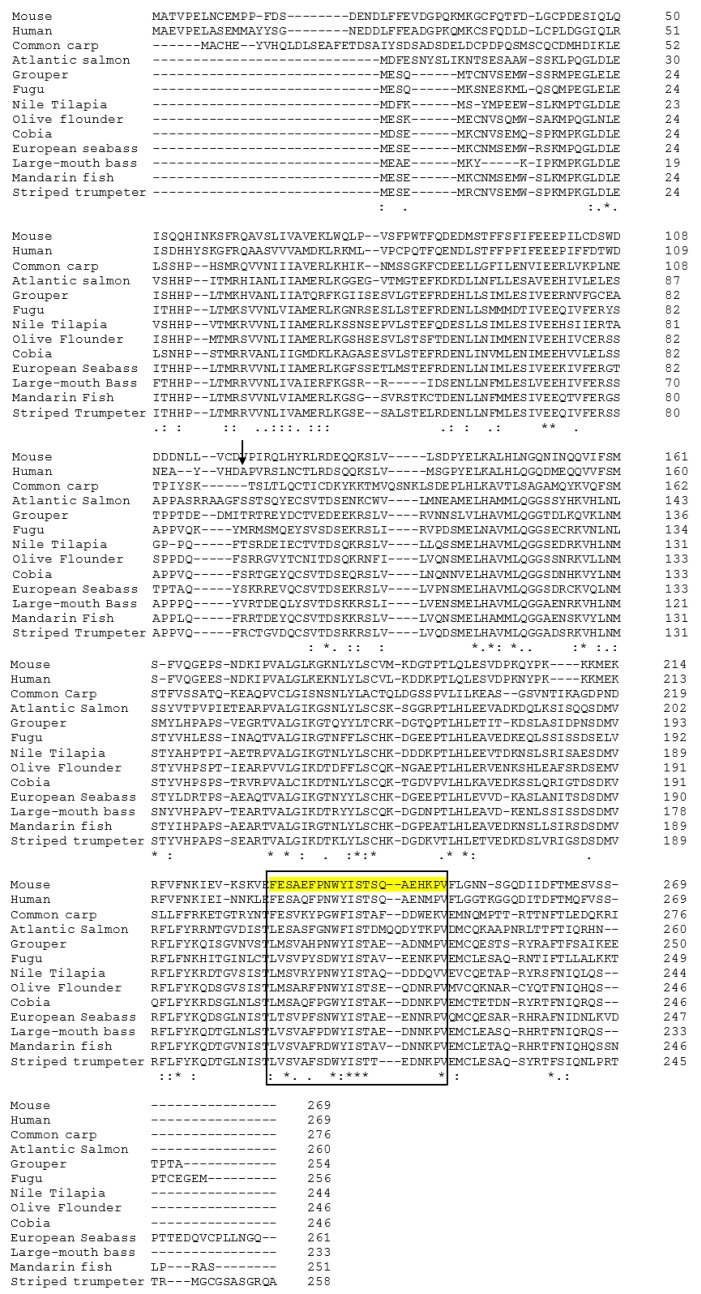
Amino acid sequence alignment of largemouth bass (*Micropterus salmoides*) IL-1β deduced protein to Mandarin perch (AAV6501.1), Striped trumpeter (ACQ99510.1), European seabass (CAC41006.1), cobia (AFV60967.1), Nile Tilapia (XP_003460673.3), Olive flounder (BAM66988.1), Fugu (NP_001267019.1), Grouper (ABV02593.1), Atlantic salmon (CAC83518.1), Common carp (BAA24538.1), mouse (AAA39276.1) and human (AAA59135.1). Arrow-aspartic region, the IL-1 signature is indicated by a black box; - - - - Sequence gaps, “*” identical residues, “:” conserved substitution, and “.” semi conserved substitution.

The domain structures of *Micropterus salmoides* (largemouth bass) IL-1β, *Siniperca chuatsi* (mandarin perch), *Latris lineata* (striped trumpeter), *Dicentrarchus labrax* (European sea bass), *Rachycentron canadum* (cobia), *Orechormis niloticus* (Nile tilapia), *Cyprinus carpio* (common carp), *Mus musculus* (mouse), and *Homosapiens* human were compared (Figure 2). The position of the IL-1 signature varied in the full length sequence of IL-1β in fish, human, and mouse. Striped trumpeter, common carp, mouse and human showed IL1_proprep domain on their full length sequence. However, the software used failed to predict the IL1_proprep domain in the rest of the analyzed species, including that of LMBIL-1β. The predicted secondary structure of the LMBIL-1β protein (Figure 3) was found to contain one helix. The generated 3D structure was found to contain single helices and also corroborated with the biological architecture of the IL-1β of other species (Figure 4). The multiple alignment of the deduced LMBIL-1β amino acid sequence and other orthologs are shown in Figure 5 and revealed many areas of strong amino acid conservation. The largemouth bass IL-1β gene lacks an aspartic acid which can be found at the cut site region of mammalian IL-1βs (Figure 5). Sequence comparisons among the putative vertebrate IL-1β amino acid sequences revealed that LMBIL-1β shared the highest amino acid identity with striped trumpeter IL-1β (73%) and mandarin perch IL-1β (73%) followed by European sea bass IL-1β (66%). The sequence identity between LMBIL-1β and common carp was lower as 35% (Table 1).

### 2.2. Phylogenetic Analysis of Teleosts IL-1β

**Figure 6:**
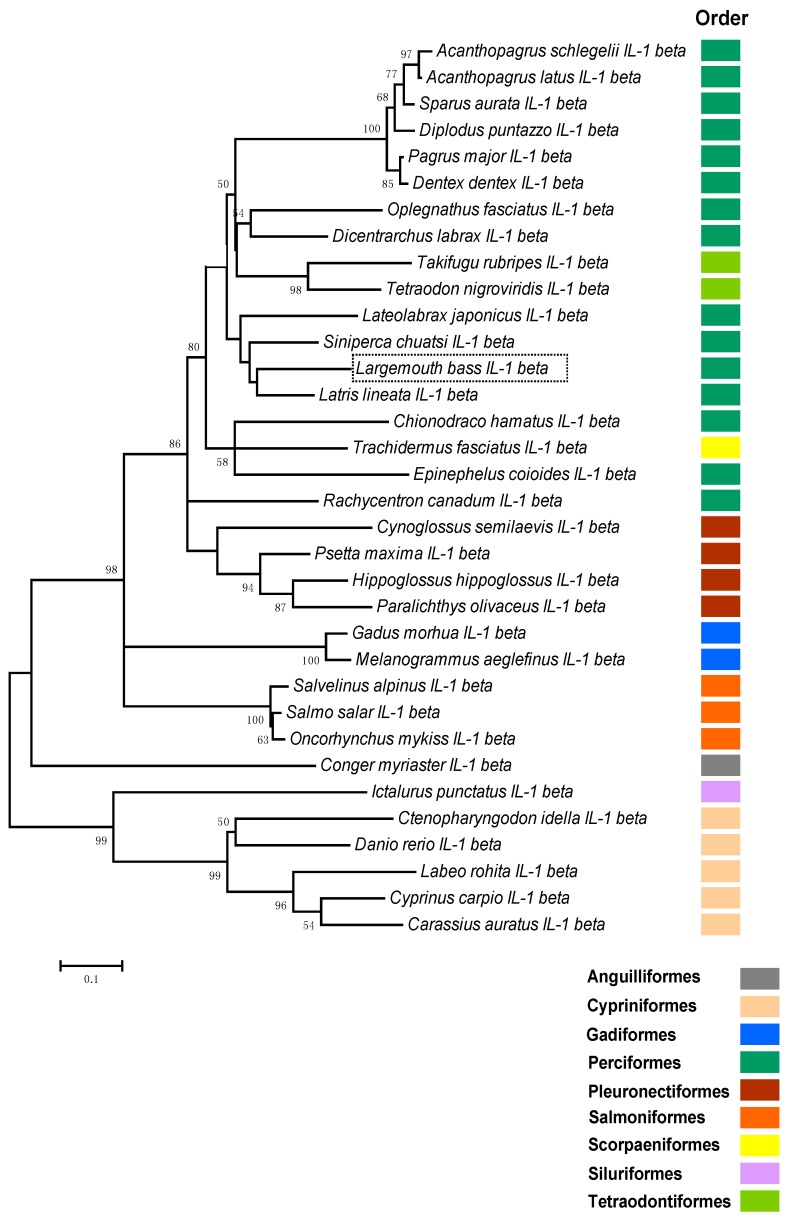
The amino acid sequences of known teleost IL-1βs were aligned with largemouth bass, *Micropterus salmoides*, IL-1β and a phylogenetic tree was constructed using the neighbor-joining method.

Thirty-three teleost species of nine orders with known IL-1β sequences were compared and phylogenetically compared with largemouth bass IL-1β using the neighbor-joining method (Figure 6). The evolution tree was divided into two groups (Figure 6). One group contained IL-1β from Cypriniformes and Siluriformes, while the other contained IL-1β from fish species belonging to Salmoniformes, Gadiformes, Perciformes, Anguilliformes, Pleuronectiformes, Scorpaeniformes, and Tetradontiformes. IL-1β from largemouth bass clustered well with its counterparts from other teleosts within the Perciformes with high bootstrap values and was closely related to *Latris lineata* and *Siniperca chuatsi*.

### 2.3. Expression Profile of LMBIL-1β in Spleen and Head Kidney after LPS and Peptidoglycan Stimulation

**Figure 7:**
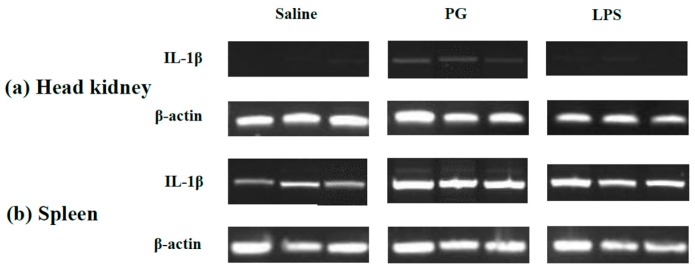
Analysis and amplification of putative largemouth bass *Micropterus salmoides* in head kidney (**a**) and spleen (**b**), IL-1β fragments resolved by RT-PCR and gel electrophoresis.

The expression of LMBIL-1β transcripts was induced by LPS or peptidoglycan (PGN) injection at 24 h (Figure 7). The injection of PGN induced IL-1β expression in the head kidney and spleen of fish. Levels of expression were higher in the spleen, but expression was also higher in the saline injected controls. LPS injection did not result in a detectable induction of LMBIL-1β in the head kidney, but did result in higher levels of LMBIL-1β expression, compared to saline injected controls, in the spleen.

### 2.4. Expression Profile of LMBIL-1β and TNF-α in Spleen and Head Kidney after Nocardia Seriolae Challenge

A melting curve analysis of the amplification products confirmed that only one PCR product, of the correct size, was amplified for IL-1β and β-actin, indicating no/undetectable contamination with genomic DNA in the cDNA material.

*Nocardia seriolae* infection significantly increased the expression of LMBIL-1β and TNF-α in a time dependent manner.

#### 2.4.1. LMBIL-1β mRNA Expression

**Figure 8:**
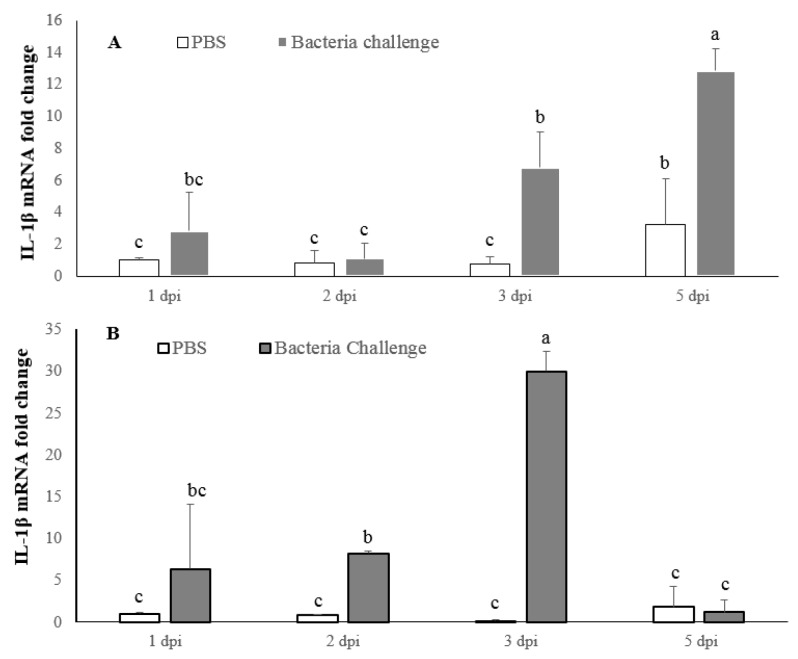
Analysis of largemouth bass, *Micropterus salmoides*, IL-1β expression in the spleen (**A**) and head kidney (**B**). The results are presented as the mean ± SD (*n* = 3) and mean values with different alphabetical letters are significantly different (*p* < 0.05).

LMBIL-1β expression was significantly increased following *N. seriolae* infection, at 3 dpi (6-fold) and 5 dpi (12-fold) post-infection (dpi) in the spleen compared to control group (Figure 8A). For the head kidney, LMBIL-1β transcripts increased significantly at 3 dpi (27-fold) and decreased sharply at 5 dpi (Figure 8B).

#### 2.4.2. TNF-α mRNA Expression

**Figure 9:**
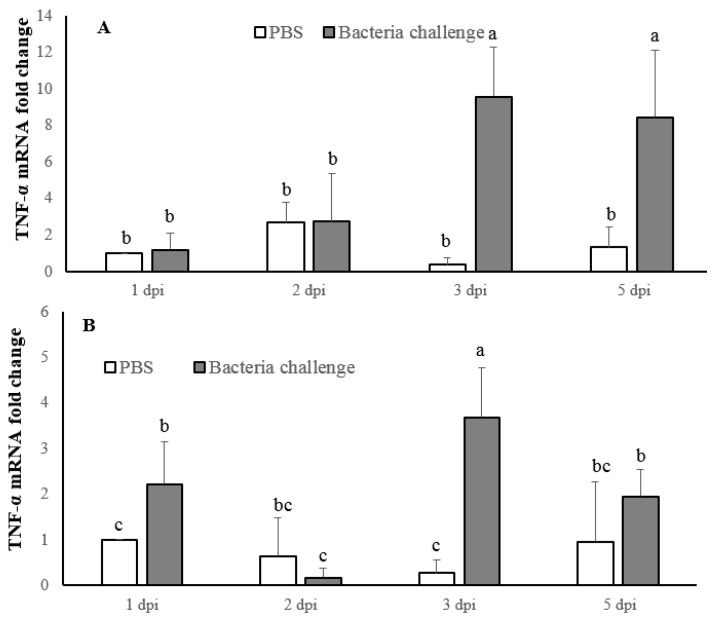
Analysis of largemouth bass, *Micropterus salmoides*, tumor necrosis factor α (TNF-α) expression in the spleen (**A**) and head kidney (**B**). The results are presented as the mean ± SD (*n* = 3) and mean values with different alphabetical letters are significantly different (*p* < 0.05).

The TNF-α expression significantly increased at 3 dpi (9-fold) and 5 dpi (8-fold) in the spleen, with no significant difference in expression levels between the two sampling points (Figure 9A). In the head kidney we observed a significant increase in expression at 1 dpi (2-fold) with the highest fold increase at 3 dpi (3-fold), while at 5 dpi the expression reduced with no significant difference with the control fish (Figure 9B).

## 3. Discussion

This study presents the full-length IL-1β cDNA sequence from the freshwater fish largemouth bass. The largemouth bass IL-1β has a high degree of homology with other known fish IL-1βs and is phylogenetically clustered within the Perciforms, for those teleosts analyzed. The sequence mostly resembles the IL-1β of the striped trumpeter, Chinese perch, and Japanese sea bass. The largemouth IL-1β mRNA is 1235 bp long. It contains 78 bp 5′-UTR and 455 bp 3′-UTR, and an ORF of 702 bp coding for 233 amino acids. The 3′-UTR sequence includes eight unstable ATTTA motifs, suggesting a tight regulation of IL-1β expression. A signature that represents the IL-1 family, [F/C/L]-x-S-[A/S/L/V]-x(2)-[P/S/K]-x(2)-[F/Y/L/I/V]-[L/I/V]-[S/C/A/T]-T-x(7)-[L/I/V/M/K] [28,29], was also conserved in largemouth bass IL-1β at amino acid positions between 194 and 214 as L-v-S-V-af-P-dw-Y-I-S-T-aednnkp-V. In common with all known teleost IL-1βs, the largemouth bass IL-1β lacks the interleukin converting enzyme (ICE) cutting site that was found in mammalian IL-1β [13,30]. Furthermore, no signal peptide was predicted in the N-terminus of the deduced largemouth bass IL-1β sequence. In our research, we used SMART software (available online: http://smart.embl-heidelberg.de/) to predict domain structure and signal peptides for IL-1β from fish and mammalian models (Figure 2). However, the program failed to detect the signal peptide for all of the tested sequences, from different organisms. Nevertheless, the signal peptide was identifiable in all analyzed protein sequences using the SignalP 4.1 server (available online: http://www.cbs.dtu.dk/services/SignalP/). The lack of an ICE cutting site and signal peptide makes the process for post-translational processing and secretion of mature IL-1β in teleost immune cells illusive.

Lipopolysaccharide (LPS) and peptidoglycan (PGN) are typical inducers of inflammatory cytokines. They are also the major components of pathogen-associated molecular patterns of Gram-negative and Gram-positive bacteria, respectively. The induction by PGN on pro-inflammatory IL-1β mRNA in largemouth bass head kidney was stronger than that of LPS in the semi-quantitative RT–PCR analysis. However, both elicitors induced a higher level of IL-1β expression in the spleen and basal expression was also observed in the control group. These results may suggest that PGN was more potent than an equal dose of LPS in stimulating head kidney IL-1β expression in vivo. What is evident is that the spleen was more sensitive than the head kidney to immune stimulation. However, the lower induction from LPS compared to PGN in largemouth bass may be due to the strain of LPS used, distinct immune cell systems, species variation, or in experimental systems.

In this study, largemouth bass injected with a virulent strain of *Nocardia seriolae* showed a significantly higher expression of IL-1β in the spleen, and kidney at 3 d post-infection. In the kidney, a down-regulation was observed at 5 d post infection. The increase in IL-1β mRNA after bacterial infection may be due to the up-regulation of gene expression as well as the proliferation and recruitment of IL-1β expressing cells in the tissues [31]. In Japanese flounder (*Paralichthys olivaceus*), the immersion challenge with *N. seriolae* suspensions induced significant IL-1β expression in the spleen and head kidney [32]. 

TNF-α was also significantly increased in the spleen and head kidney during bacterial infection. TNF-α, secreted mainly by macrophages/monocytes and T lymphocytes helps in the regulation of inflammation and cellular immune responses [33,34]. The expression of TNF-α in largemouth bass supports the finding that the TNF-α gene is significantly expressed at early stages of infection [35]. The relation between IL-1β and TNF-α expression after *N. seriolae* challenge is not unexpected [31]. 

Fish nocardiosis induces chronic disease, exhausting fish and making them more susceptible to other bacterial, viral or parasitic infections. Either external or internal lesions of infected fish make them unmarketable. Since IL-1β is effective in activating host defenses by activating B and T-cells, it is also regarded as an immuno-modulator. The recombinant IL-1β has been shown to act as a vaccine adjuvant for carp, increasing agglutinating activities of serum against *Aeromonas hydrophila* [36]. The immuno-adjuvant effects have also been observed in sea bass (*Dicentrarchus labrax*) and rainbow trout (*Oncorhynchus mykiss*) [37,38]. Therefore, recombinant IL-1β with bioactivity may act as a potent adjuvant with antigens or inactivated bacteria.

In summary, the IL-1β transcript of largemouth bass was cloned and the expressional profiles in immune tissues following *N. seriolae* challenge were analyzed. Sequence homology revealed some similarity with the IL-1βs of the Perciform teleost. The IL-1β expression in the spleen was stronger than that in the head kidney of challenged fish, based on a time-course evaluation. IL-1β expression can serve as an immune index and IL-1β can be a potential adjuvant in assessment of disease or in development of a vaccine.

## 4. Materials and Methods

### 4.1. Animal Maintenance

For acclimatization, largemouth bass (*Micropterus salmoides*), without any specific pathogenic infection, were maintained in an indoor facility and fed with Bass feed No. 4, (Chuen Shin feed manufacturer, Pingtung, Taiwan). Whenever the fish were handled, 2-phenoxyethanol was used as the anesthetic agent. For this experiment and animal use, approval was obtained from the Center for Research Animal Care and Use Committee of the National Pingtung University of Science and Technology under protocol no # 101-027 dated 19 March 2012.

### 4.2. Primer Design and RT-PCR to Isolate Partial IL-1β Sequence from Largemouth Bass

The IL-1β degenerated primer FIL1BF and FIL1BR (Table 2 and Table 3) were designed based on the conserved homology of fish IL-1β sequences that are available in public databases. Table 2 and Table 3 present the primers used in this study and the corresponding sizes of the amplicons. RNA from the spleen was reverse-transcribed using mMLV reverse-transcriptase (Promega, Madison, WI, USA) with oligo-dT primer, according to the manufacturer’s instruction. The resultant cDNA was used as a template in PCR amplification. The PCR reaction involves 1.5–2 mM MgCl_2_, 10 mM Tris-HCl, 40 mM KCl, 200–250 μM dNTP, 0.5–1 U GenTaq DNA polymerase (GM Biolab) and 100–200 ng first-strand cDNA. Each set of primers (0.5–1 μM) included in the reaction mixture was added to yield a final volume of 50 μL. The thermal cycling program was preheated at 95 °C for 10 min; 30 cycles of denaturation at 95 °C for 30 s, annealing at 57 °C for 30 s and extension at 72 °C for 1 min, and a final extension at 72 °C for 7 min. Amplified fragments were analyzed by 2% agarose gel electrophoresis that contained 0.01% ethidium bromide. Gels were photographed under UV irradiation.

### 4.3. Sequencing and Full-Length cDNA Cloning

PCR-amplified fragments with desired sizes were cloned into the vector pCR II/TOPO (Invitrogen, Carlsbad, CA, USA), according to the manufacturer’s instructions. The competent *E. coli* DH5α (Genemark, Taiwan) was chemically transformed by constructed plasmids under heat shock at 42 °C for 30 s. Following the addition of super optimal broth S.O.B. medium and subsequent 1 h of incubation at 37 °C with agitation, bacterial suspension was plated onto the Luria Bertani (LB) agar containing X-gal. The plate was then incubated at 37 °C for 14 h. Positive clones, identified by blue/white screening were then checked by PCR. The clones that contained the plasmid with appropriate inserts were then sub-cultured and sent to a biotech company for sequencing (Tri-Bio Inc., Taiwan). Obtained sequence data were blasted against an NCBI database to find identities and similarities with other known IL-1β. 

To resolve the full-length mRNA, first-strand cDNA that was derived from PGN-stimulated largemouth bass spleen was used. The SMARTer RACE cDNA amplification kit (Clontech, Mountain View, CA, USA) was used for both 5′ and 3′ RACE cDNA sequences. The 5′ RACE was performed with primer LMBIL-1b_R and Nested Universal Primer A (NUP, Clontech, Mountain View, CA, USA) (Table 2) for 25 cycles of 94 °C for 30 s, 68 °C for 30 s, and 72 °C for 2 min, which were followed by 15 cycles of 94 °C for 30 s, 58 °C for 30 s, and 72 °C for 2 min, and an extension at 72 °C for 10 min in the final step. The reactions were analyzed by gel electrophoresis to characterize the amplicons. The 3′ RACE was conducted with primer LMBIL1B3R1 and Universal Primer Mix (Table 1) (UPM, Clontech, Mountain View, CA, USA) for 25 cycles at 94 °C for 30 s, 68 °C for 30 s, and 72 °C for 2 min. After checking the amplified fragments of interest by electrophoresis, the products thus obtained were ligated into pCR II/TOPO vector for sequencing.

### 4.4. In-Silico Analysis of Largemouth Bass IL-1β Sequence

The ExPASy proteomic tool (available online: http://expasy.org/tools/) was used to predict molecular weight (kDa), pI of protein sequence. SMART software (available online: http://smart.embl-heidelberg.de/) was used to determine protein domains. The protein prediction PSIPRED server (available online: http://bioinf.cs.ucl.ac.uk/psipred/psiform.html) was used to determine secondary structure and phyre2 server for 3D structure [39].

### 4.5. Phylogenetic Analysis

The complete amino acid sequences of known teleost IL-1βs were aligned with largemouth bass, *Micropterus salmoides* IL-1β using ClustalW (available online: http://ebi.ac.uk/clustalw2) and a phylogenetic tree was constructed using the CLUSTAL method of MegAlign (DNAstar 5.0, DNASTAR Inc., Madison, WI, USA). Support for the species groupings was determined by bootstrap analysis and using 1000 re-samplings.

### 4.6. Fish IL-1β Induction and RNA Extraction

Three tanks that each contained three fish were set up with water circulation and aeration systems. Fish were anesthetized and injected intraperitoneally with peptidoglycan (PGN) and lipopolysaccharide (LPS) (Sigma, St. Louis, MO, USA) at 0.2 mg/fish. After 24 h, the head kidney and spleen tissues were collected and stored in RNA-Keeper (Promega, Madison, WI, USA) at 4 °C for subsequent RNA extraction. Aliquot tissue sample was subjected to RNA extraction by Trizol^®^ (Invitrogen, Carlsbad, CA, USA) according to the manufacturer’s instruction. The RNA quality was confirmed using the ratios A260/280 and A260/230 (Thermo fisher scientific, Waltham, MA, USA), and then frozen at −80 °C until use. cDNA was synthesized as described previously.

### 4.7. IL-1β Expressional Profiling by RT-PCR

The expression of IL-1β, in PGN-LPS stimulated head kidney and spleen was analyzed by RT–PCR using largemouth bass specific IL-1β primers, LMBIL-1b_F and LMBIL-1b_R (Table 2 and Table 3). The thermocycling conditions for the RT–PCR reactions were as follows: pre-heating at 94 °C for 5 min, 30 cycles of 94 °C for 30 s, 58 °C for 30 s and 72 °C for 1 min, and a final extension step at 72 °C for 10 min. The β-actin transcripts were amplified as an internal control using primers β-actin375F and β-Actin375R (Table 2 and Table 3) with pre-heating at 94 °C for 5 min, 25 cycles at 94 °C for 30 s, 62 °C for 30 s and 72 °C for 45 s, and a final extension step at 72 °C for 10 min. The PCR products were analyzed by gel electrophoresis for expressional profiling and comparisons.

### 4.8. Isolation, Cultivation and Challenge with Nocardia Seriolae

*Nocardia seriolae* was isolated from striped bass and was found to be highly virulent in farmed fish. The species was identified by analytical profile index- Zymogen (API ZYM) and 16S rDNA sequencing [40]. The *N. seriolae* were prepared in brain heart infusion (BHI) broth for five days at 25 °C, adjusted OD = 1.0 (610 nm) and enumerated the concentration using serial dilution before the challenge test. Fifteen fish were anesthetized and intraperitoneally injected with 1.0 × 10^6^ cfu *N. seriolae* that was suspended in 100 μL PBS (pH 7.2). The second treatment with 15 fish received only PBS (pH 7.2) and served as a control. After the fish were returned to the observation tanks, samples were taken at 1, 2, 3 and 5 days post infection (dpi). Three fish from the challenge and control groups (*n* = 3) were sampled at each time point, respectively. The head kidney and spleen tissue were dissected and stored in RNA-Keeper (Promega) at 4 °C for subsequent RNA extraction.

### 4.9. Real Time Polymerase Chain Reaction

Quality and quantity of extracted RNA was assessed as described previously. A total of 2 µg of total RNA was used to synthesize cDNA using M-MLV reverse transcriptase (Promega). The expression of IL-1β, and TNF-α in *Nocardia seriolae* infected tissue were analyzed. β-actin served as the internal control. Thermal gradient feature CFX96 system (Bio-Rad Laboratories, Hercules, CA, USA) was used to determine the optimal annealing temperature for IL-1β and TNF-α primers. The real-time PCR program was 95 °C for 3 min, followed by 40 cycles of 95 °C for 15 s, 58 °C (IL-1β), 60 °C (TNF-α) for 15 s and 72 °C for 35 s. The reaction (*n* = 3) was carried out using iQSYBR Green Supermix (Bio-Rad Laboratories). A CFX Manager Software package (Bio-Rad Laboratories) was used to calculate the gene expression using the 2^−ΔΔ*C*t^ method [41]. 

### 4.10. Statistical Analysis

SPSS 16.0 software was used to analyze the data. One-way ANOVA (analysis of variance) and Duncan’s test at 0.05 were used to test for level of significance.

## Figures and Tables

**Table 1:** Pairwise comparison of the IL-1β amino acid sequence of largemouth bass with IL-1β of other fish species.

Species Name	Amino Acid Identity (%)
Mandarin perch	73
Striped trumpeter	73
Striped beak fish	65
European sea bass	66
Japanese sea bass	65
Nile tilapia	63
Gilthead seabream	58
Cobia	61
Orange spotted grouper	55
Fugu	61
Atlantic salmon	53
Rainbow trout	54
Common carp	35

**Table 2:** Primers used for cloning.

Primer Name	Primer Sequence (5′–3′)	Application
FIL1BF	TGGAMYTKGAGATTDCMCA	Partial cloning
FIL1BR	AAAYCKYACCATGTCGCTG	
Universal Primer Mix (UPM)	Long 0.2 μM CTAATACGACTCACTATAGGGCAAGCAGTGGTATCAACGCAGAGT	RACE
	Short 0.4 μM CTAATACGACTCACTATAGGGC	
Nested Universal Primer Mix (NUP)	AAGCAGTGGTATCAACGCAGAGT	
LMBIL1B3R1	GCATCAAAGACACACGTTACTACCTGTC	
LMBIL-1b_F	TGGACTTGGAGATTGCCCA	q-PCR
LMBIL-1b_R	AAACCGCACCATGTCGCTG	

**Table 3:** Primer name, sequence, target gene, and their application used in the present study.

Primer Name	Primer Sequence (5′–3′)	Target Gene	Application
β-Actin375F	CCACCACAGCCGAGAGGGAA	β-actin	q-PCR
β-Actin375R	TCATGGTGGATGGGGCCAGG		
IL-1βF	TTGCCATAGAGAGGTTTA	IL-1β	
IL-1βR	ACACTATATGCTCTTCCA		
TNFα-F	CTAGTGAAGAACCAGATTGT	TNF-α	
TNFα-R	AGGAGACTCTGAACGATG		

## References

[1] Dinarello C.A. (1988). Biology of interleukin 1. FASEB J..

[2] Dinarello C.A. (2010). IL-1: Discoveries, controversies and future directions. Eur. J. Immunol..

[3] Dinarello C.A. (1996). Biologic basis for interleukin-1 in disease. Blood.

[4] Howard A.D., Kostura M.J., Thornberry N., Ding G.J., Limjuco G., Weidner J., Salley J.P., Hogquist K.A., Chaplin D.D., Mumford R.A. (1991). IL-1-converting enzyme requires aspartic acid residues for processing of the IL-1β precursor at two distinct sites and does not cleave 31-kDa IL-1 α. J. Immunol..

[5] Dinarello C.A. (2002). The IL-1 family and inflammatory diseases. Clin. Exp. Rheumatol..

[6] Lopez-Castejon G., Brough D. (2011). Understanding the mechanism of IL-1β secretion. Cytokine Growth Factor Rev..

[7] Weber A., Wasiliew P., Kracht M. (2010). Interleukin-1 (IL-1) pathway. Sci. Signal..

[8] Clem L.W., Sizemore R.C., Ellsaesser C.F., Miller N.W. (1985). Monocytes as accessory cells in fish immune responses. Dev. Comp. Immunol..

[9] Sigel M.M., Hamby B.A., Huggins E.M. (1986). Phylogenetic studies on lymphokines. Fish lymphocytes respond to human IL-1 and epithelial cells produce an IL-1 like factor. Vet. Immunol. Immunopathol..

[10] Verburg-van Kemenade B.M., Weyts F.A., Debets R., Flik G. (1995). Carp macrophages and neutrophilic granulocytes secrete an interleukin-1-like factor. Dev. Comp. Immunol..

[11] Weyts F.A.A., Rombout J.H.W.M., Flik G., Verburg-Van Kemenade B.M.L. (1997). A common carp (*Cyprinus carpio*) leucocyte cell line shares morphological and functional characteristics with macrophages. Fish Shellfish Immunol..

[12] Secombes C.J., Bird S., Cunningham C., Zou J. (1999). Interleukin-1 in fish. Fish Shellfish Immunol..

[13] Zou J., Grabowski P.S., Cunningham C., Secombes C.J. (1999). Molecular cloning of interleukin 1β from rainbow trout *Oncorhynchus mykiss* reveals no evidence of an ice cut site. Cytokine.

[14] Watzke J., Schirmer K., Scholz S. (2007). Bacterial lipopolysaccharides induce genes involved in the innate immune response in embryos of the zebrafish (*Danio rerio*). Fish Shellfish Immunol..

[15] Morrison R.N., Young N.D., Nowak B.F. (2012). Description of an Atlantic salmon (*Salmo salar* L.) type II interleukin-1 receptor cDNA and analysis of interleukin-1 receptor expression in amoebic gill disease-affected fish. Fish Shellfish Immunol..

[16] Vojtech L.N., Scharping N., Woodson J., Hansen J.D. (2012). Roles of inflammatory caspases during processing of zebrafish interleukin-1β in *Francisella noatunensis* infection. Infect. Immun..

[17] Morrison R.N., Zou J., Secombes C.J., Scapigliati G., Adams M.B., Nowak B.F. (2007). Molecular cloning and expression analysis of tumor necrosis factor-α in amoebic gill disease (AGD)-affected Atlantic salmon (*Salmo salar* L.). Fish Shellfish Immunol..

[18] Bo Y.X., Song X.H., Wu K., Bo H., Sun B.Y., Liu Z.J. (2015). Characterization of interleukin-1β as a proinflammatory cytokine in grass carp. Fish Shellfish Immunol..

[19] Kudo T., Hatai K., Seino A. (1988). *Nocardia seriolae* sp. nov. Causing nocardiosis of cultured fish. Int. J. Syst. Evol. Microbiol..

[20] Itano T., Kawakami H., Kono T., Sakai M. (2006). Live vaccine trials against nocardiosis in yellowtail *Seriola quinqueradiata*. Aquaculture.

[21] Snieszko S.F., Bullock G.L., Dunbar C.E., Pettijohn L.L. (1964). Nocardial infection in hatchery-reared fingerling rainbow trout (*Salmo gairdneri*). J. Bacteriol..

[22] Chen S.C., Tung M.C., Tsai W.C. (1989). An epizootic in Formosa snake-head fish, *Channa maculata* Lacepede, caused by *Nocardia asteroides* in fresh water pond in southern Taiwan. COA Fish Ser..

[23] Chen S.C., Tung M.C. (1991). An epizootic in largemouth bass, *Micropterus salmoides*, Lacepede caused by *Nocardia asteroides* in freshwater pond in southern Taiwan. J. Chin. Soc. Vet. Sci..

[24] Wang G.L., Xu Y.J., Jin S., Zhu J.L., Yuan S.P. (2007). Nocardiosis in snakehead, *Ophiocephalus argus* cantor. Aquaculture..

[25] Chen S.C., Lee J.L., Lai C.C., Gu Y.W., Wang C.T., Chang H.U., Tsai K.H. (2000). Nocardiosis in sea bass, *Lateolabrax japonicus*. J. Fish Dis..

[26] Wang G.L., Yuan S.P., Jin S. (2005). Nocardiosis in large yellow croaker, *Larimichthys crocea* (Richardson). J. Fish Dis..

[27] Wang P.C., Chen S.D., Tsai M.A., Weng Y.J., Chu S.Y., Chern R.S., Chen S.C. (2009). *Nocardia seriolae* infection in the three striped tigerfish, *Terapon jarbua* (Forsskål). J. Fish Dis..

[28] Bird S., Zou J., Wang T., Munday B., Cunningham C., Secombes C.J. (2002). Evolution of interleukin-1β. Cytokine Growth Factor Rev..

[29] Lee D.S., Hong S.H., Lee H.J., Jun L.J., Chung J.K., Kim K.H., Jeong H.D. (2006). Molecular cDNA cloning and analysis of the organization and expression of the IL-1β gene in the Nile tilapia, *Oreochromis niloticus*. Comp. Biochem. Physiol. A.

[30] Fujiki K., Shin D.H., Nakao M., Yano T. (2000). Molecular cloning and expression analysis of carp (*Cyprinus carpio*) interleukin-1 β, high affinity immunoglobulin E Fc receptor γ subunit and serum amyloid A. Fish. Shellfish. Immunol..

[31] Mulder I.E., Wadsworth S., Secombes C.J. (2007). Cytokine expression in the intestine of rainbow trout (*Oncorhynchus mykiss*) during infection with *Aeromonas salmonicida*. Fish Shellfish Immunol..

[32] Tanekhy M., Matsuda S., Itano T., Kawakami H., Kono T., Sakai M. (2009). Expression of cytokine genes in head kidney and spleen cells of Japanese flounder (*Paralichthys olivaceus*) infected with *Nocardia seriolae*. Vet. Immunol. Immunopathol..

[33] Zou J., Clark M.S., Secombes C.J. (2003). Characterization, expression and promoter analysis of an interleukin 10 homologue in the puffer fish, *Fugu rubripes*. Immunogenetics.

[34] Laing K.J., Wang T., Zou J., Holland J., Hong S., Bols N., Hirono I., Aoki T., Secombes C.J. (2001). Cloning and expression analysis of rainbow trout *Oncorhynchus mykiss* tumor necrosis factor-α. Eur. J. Biochem..

[35] Savan R., Sakai M. (2006). Genomics of fish cytokines. Comp. Biochem. Physiol..

[36] Yin Z., Kwang J. (2000). Carp interleukin-1 β in the role of an immuno-adjuvant. Fish Shellfish Immunol..

[37] Buonocore F., Mazzini M., Forlenza M., Randelli E., Secombes C.J., Zou J., Scapigliati G. (2004). Expression in *Escherichia coli* and purification of sea bass (*Dicentrarchus labrax*) interleukin 1β, a possible immunoadjuvant in aquaculture. Mar. Biotechnol..

[38] Hong S., Secombes C.J. (2009). Two peptides derived from trout IL-1β have different stimulatory effects on immune gene expression after intraperitoneal administration. Comp. Biochem. Physiol. B. Biochem. Mol. Biol..

[39] Kelley L.A., Sternberg M.J.E. (2009). Protein structure prediction on the Web: A case study using the Phyre server. Nat. Protoc..

[40] Itano T., Kawakami H., Kono T., Sakai M. (2005). Detection of fish nocardiosis by loop-mediated isothermal amplification. J. Appl. Microbiol..

[41] Livak K.J., Schmittgen T.D. (2001). Analysis of relative gene expression data using real time quantitative PCR and the 2 ^(−∆∆*C*t)^ method. Methods.

